# Influences of Vitamin D and Iron Status on Skeletal Muscle Health: A Narrative Review

**DOI:** 10.3390/nu14132717

**Published:** 2022-06-29

**Authors:** Marni E. Shoemaker, Owen F. Salmon, Cory M. Smith, Maria O. Duarte-Gardea, Joel T. Cramer

**Affiliations:** 1 School of Health and Consumer Sciences, South Dakota State University, Brookings, SD 57007, USA; 2 Department of Health, Human Performance, and Recreation, Baylor University, Waco, TX 76798, USA; owen_salmon1@baylor.edu (O.F.S.); cory_m_smith@baylor.edu (C.M.S.); 3 Department of Public Health Sciences, The University of Texas at El Paso, El Paso, TX 79968, USA; moduarte@utep.edu; 4 Department of Kinesiology, The University of Texas at El Paso, El Paso, TX 79968, USA; jtcramer@utep.edu

**Keywords:** animal food sources, iron, muscle function, muscle mass, muscle strength, vitamin D

## Abstract

There is conflicting evidence of the roles vitamin D and iron have in isolation and combined in relation to muscle health. The purpose of this narrative review was to examine the current literature on the roles that vitamin D and iron have on skeletal muscle mass, strength, and function and how these nutrients are associated with skeletal muscle health in specific populations. Secondary purposes include exploring if low vitamin D and iron status are interrelated with skeletal muscle health and chronic inflammation and reviewing the influence of animal-source foods rich in these nutrients on health and performance. PubMed, Scopus, SPORT Discus, EMBAE, MEDLINE, and Google Scholar databases were searched to determine eligible studies. There was a positive effect of vitamin D on muscle mass, particularly in older adults. There was a positive effect of iron on aerobic and anaerobic performance. Studies reported mixed results for both vitamin D and iron on muscle strength and function. While vitamin D and iron deficiency commonly occur in combination, few studies examined effects on skeletal muscle health and inflammation. Isolated nutrients such as iron and vitamin D may have positive outcomes; however, nutrients within food sources may be most effective in improving skeletal muscle health.

## 1. Introduction

Many nutrients are essential for skeletal muscle health including vitamin D and iron. These nutrients have been well-established to play a role in improving muscular strength, muscle mass, and muscle function in various populations such as youth, athletes, and older adults [1,2,3,4,5,6,7,8]. Together, muscular strength, muscle mass, and muscle function are important components that make up “skeletal muscle health” and will be the primary focus within this review. Beyond the role that these nutrients have on skeletal muscle health, emerging literature has reported a link between vitamin D action on pro-inflammatory cytokines and mechanisms behind iron regulation [9,10,11], which may physiologically play a role in skeletal muscle health. 

Vitamin D plays a role in the body’s inflammatory response through activation and differentiation of immune and inflammatory cells [12,13]. Furthermore, adequate levels of vitamin D has been shown to decrease the production of pro-inflammatory cytokines such as IL-12, interferon gamma (IFN-γ), IL-6, TNF-α, IL-17, IL-9 and increase the production of anti-inflammatory cytokines such as IL-4, IL-5, and IL-10 [14,15,16,17,18]. Although many studies utilize different definitions, 25(OH)D levels <20 ng·mL^−1^ (50 nmol·L^−1^) have been defined as deficient and levels 30–60 ng·mL^−1^ (75–150 nmol·L^−1^) are defined as insufficient. Levels of >75 nmol·L^−1^ are thought to be optimal for health [19]; however, recent evidence has suggested that levels of at least 100 nmol·L^−1^ may be necessary for older adults [20]. Although the Recommended Daily Allowance (RDA) for vitamin D for individuals between ages 9–70 years is 600 IU·d^−1^ [19], intakes between 1500 and 2000 IU·d^−1^ have been thought necessary to increase blood concentrations to above deficient levels [21]. Due to insufficient intake [6,22], lack of exposure to sunlight [23,24], skin pigmentation [25,26], and inhibition of absorption [27,28,29,30,31,32], many individuals have a low vitamin D status. Older adults, in particular, have decreased vitamin D production from sun exposure due to lower 7-dehydrocholesterol in the skin, thus compounded the risk in this population [33]. Additionally, Vitamin D is linked to the regulation of iron metabolism through its effects on hepcidin, indicating that low vitamin D levels may consequently result in iron deficiency and/or anemia [9,11,34,35]. Therefore, maintaining adequate vitamin D and iron status may be necessary for clinically related outcomes such as healthy growth, proper bone and tissue development, and a reduction in incidences of sarcopenia and various other chronic diseases [36,37,38,39]. Furthermore, with more research suggesting a link between skeletal muscle health and chronic inflammation, identifying methods to mitigate inflammation may be essential for the optimization of muscle health throughout the lifespan. 

Nutritional deficiencies are common in athletes, youth, and older adults, with vitamin D and iron being common micronutrients that are deficient. In fact, prevalence of low vitamin D status ranges from 17.4 to 87% in older adults [22,40], from 34 to 75% in adult and youth athletes [41,42], and from 21 to 49% in normal to severely obese youth [43], indicating the severity of this issue in multiple populations. The prevalence of low iron status ranges from 11 to 33% in older adults [44,45], 46 to 86% in youth and adult athletes [46,47], and 4 to 14% in youth [48]. Additionally, iron deficiency and anemia have been linked to low vitamin D status [49,50], suggesting that a combination of these nutritional deficiencies causes compounded risk to muscle health and inflammation. Since the combination of vitamin D deficiency and low iron status is prevalent in many populations including older adults [22,40,44,45,51], youth [52,53,54,55], and athletes [47,56,57], this highlights the need to examine a potential interaction and symbiotic relationship between these nutrients, particularly regarding inflammation and muscle health. 

Adequate consumption of vitamin D and iron may be key in enhancing muscle mass, strength, and performance. Animal-source foods are abundant in vitamin D and iron, which may help individuals reach optimal, bioavailable intakes of these nutrients to support skeletal muscle health [58,59,60]. Thus, we aim to investigate the potential impacts of vitamin D, iron, and animal-source foods containing these nutrients on skeletal muscle health and chronic inflammation.

The purpose of this narrative review was to examine the current literature on the roles that vitamin D and iron have on skeletal muscle mass, muscle strength, and muscle function and how these nutrients are associated with skeletal muscle health in specific populations such as youth, athletes, and older adults. Secondary purposes include (a) exploring if low vitamin D and iron statuses are interrelated with skeletal muscle health and chronic inflammation and (b) reviewing the influence of animal-source foods rich in these nutrients on health and performance. Furthermore, this narrative review aimed to identify gaps in the literature for future research focused on health benefits of animal-source foods targeted at specific populations such as youth, athletes, and older adults. 

## 2. Materials and Methods

A review of the literature was performed using PubMed, Scopus, SPORT Discus, EMBAE, OVID MEDLINE, and Google Scholar databases. Cross-sectional, observational, longitudinal, and experimental studies that examined nutritional status (i.e., serum or plasma concentrations) and/or dietary intake of vitamin D and iron along with measurement of health outcomes related to skeletal muscle and inflammation were included in the review. Publications after 2010 were included, and articles were only included as reference sources if published in peer-reviewed journals in the English language. Relevant articles were identified and checked for eligibility by two independent researchers. Relevant articles were categorized according to the specific aims of the narrative literature review: (1) Role of Vitamin D on Skeletal Muscle Health, (2) Role of Iron on Skeletal Muscle Health, (3) Interrelationship between Vitamin D, Iron and Chronic Inflammation, (4) Animal Food Sources as a Strategy to Improve Skeletal Muscle Health. 

## 3. Discussion

### 3.1. Vitamin D and Skeletal Muscle Health

#### 3.1.1. Vitamin D and Skeletal Muscle Physiology

Vitamin D is a fat-soluble prohormone that provides key functions in multiple endocrine and autocrine processes throughout the body. Vitamin D can be obtained by both food sources and solar ultraviolet B (UVB) of 290–315 nm and in conjunction, is utilized to synthesize cholecalciferol, or vitamin D_3_ [61,62]. This production of vitamin D is dependent upon the solar elevation in which vitamin D is predominately produced when the elevation angle is greater than 45 degrees [63]. Once synthesized in the skin from sunlight or absorbed from food, vitamin D_3_ binds to vitamin D-binding protein (VDBP) and is transferred to the liver where it is converted to 25-hydroxycholecalciferol (25(OH)D) and transported to the kidney for further processing. Within the kidney, 25(OH)D is activated to the form of 1,25-dihydroxycholecalciferol [1,25(OH)_2_D], or calcitriol [64,65,66]. This active form can then perform functions such as calcium and phosphate regulation through the binding of vitamin D receptor (VDR). Vitamin D receptor is present on many body tissues including skeletal muscle, intestines, myocardium, bone, nervous system, as well as immune cells, indicating inadequate vitamin D effects multiple tissues within the body, resulting in a potential link to multiple pathological diseases including cardiovascular disease, inflammatory conditions, and respiratory illness [67,68,69,70,71]. 

The mechanism for the role vitamin D has on skeletal muscle involves VDR expression found in skeletal muscle cells [72,73,74]. Expression of VDR in the nucleus of skeletal muscle cells is necessary for vitamin D uptake [75], and reduced VDR concentrations have affected the contractility of muscle cells and may affect skeletal muscle repair and recovery [76,77]. Animal studies have demonstrated that mice without the VDR gene had smaller muscle fibers, lower body size and weight, and impaired movement compared to mice with VDR gene [78]. Additionally, VDR concentrations have been shown to increase after supplementation of 1,25(OH)_2_D_3_ and 25(OH)D_3_ in muscle cells, which was suggested to be linked to muscle cell regeneration [79]. 

Further support for the importance of VDR in skeletal muscle health was demonstrated in human studies. Expression of VDR primarily has been found to be located on fast-twitch muscle fibers [80], and interestingly, it has been identified that fast-twitch muscle fibers following vitamin D supplementation [81]. Thus, this provides support for the importance of VDR concentration, related to vitamin D status, for improving skeletal muscle health in humans. Supplementation with vitamin D_3_ in vitamin D insufficient females resulted increased VDR concentration [82], indicating that adequate VDR and vitamin D concentrations can support muscle fiber growth. Additionally, vitamin D actions on skeletal muscle through VDR may also influence calcium regulation and muscle contraction, anabolic or growth pathways, oxidative phosphorylation and mitochondrial function, and muscle inflammation [83].

#### 3.1.2. Vitamin D Status and Skeletal Muscle Health

Vitamin D mechanistically appears to influence skeletal muscle health. Therefore, this effect on skeletal muscle health in conjunction with a high prevalence of vitamin D deficiency in certain populations such as youth, athletes, and older adults warrants an examination of the associations between vitamin D and measurements of muscle mass, muscle strength, and muscle function. Table 1 reports studies examining associations between vitamin D status and skeletal muscle health. Many studies in athletic populations reported associations between concentrations of 25(OH)D and measurements of performance [84,85,86,87,88]; however, other studies reported no associations between vitamin D and performance outcomes [89,90]. Koundorakis et al. reported correlations between serum 25(OH)D concentrations and tests of anaerobic and aerobic performance. These included moderate to high positive associations between serum 25(OH)D and jumping ability and VO_2_max (r = 0.394–0.740) and moderate negative associations between serum 25(OH)D and sprint times (r = −410–−0.649) during soccer pre- and post-season [84]. However, in male hockey players, there were no associations between serum 25(OH)D and aerobic exercise variables determined during a graded exercise test [89]. Forney et al. reported a positive association between serum 25(OH)D and VO_2_max (r = 0.360) but not with tests of anaerobic performance or muscle strength [90]. Additionally, submaximal aerobic performance and aerobic power was better in athletes with higher serum 25(OH)D levels compared to those with levels considered deficient (<35 ng·mL^−1^ and <30 ng·mL^−1^, respectively) [87,88]. Peak torque was found to be 12–17% higher in those with higher serum 25(OH)D levels [85,86]. These results suggest that while evidence supporting a positive association between vitamin D and athletic performance is inconclusive, many studies suggest that vitamin D levels are moderately related to performance. It is important to note that a majority of the studies were performed in young adult male athletes with varying tests of performance. Future studies inclusive of male and female athletes from a variety of athletic backgrounds is necessary.

In older adults, vitamin D showed consistent low to moderate relationships with muscle mass [91,92,96], muscle strength [4,20,91,93,94,95] and muscle function [4,20,92,94,95,97], with only a few of the studies reviewed finding no associations with skeletal muscle health. For example, while Conzade et al. reported a positive relationship between serum 25(OH)D concentrations and changes in muscle mass and an inverse relationship with time to complete TUG, there were no relationships with handgrip strength and gait speed [96]. Furthermore, Vaes et al. reported that older adults with serum 25(OH)D levels <50 nmol·L^−1^ and 50–75 nmol·L^−1^ had lower scores for tests of muscle function compared to those with levels >75 nmol·L^−1^ but observed no relationships with tests of muscle strength [97] (Table 1). Similarly, in youth, positive relationships were consistently reported with tests of anaerobic (broad jump, vertical jump, sprints) performance and aerobic performance (estimated VO_2_max) [98,99,100,102] (Table 1). However, there were mixed results with tests of muscle strength and serum 25(OH)D concentrations [101,103,104]. These results indicate that vitamin D levels are impactful on skeletal muscle mass and function across the lifespan, but the effects on muscle strength is inconclusive. In addition to these findings, it is reported that 25(OH)D metabolite accumulates in skeletal muscle cells, suggesting that maintaining muscle mass can also play a role in preserving vitamin D status in times when deficiency may become prevalent, suggesting that skeletal muscle health may have an influence on vitamin D levels [105,106].

#### 3.1.3. Vitamin D Interventions and Skeletal Muscle Health

Multiple studies have examined the effects of vitamin D supplementation on muscle mass, strength, and function, typically with doses ranging from 1000 to 4000 IU·d^−1^ over 4–12 weeks to over 60,000 IU·week^−1^ for up to 4 months (Table 2). Contrasting results of the effects vitamin D supplementation has on muscle health have been reported, potentially due to the large variety in dosage, type of vitamin D supplementation, duration of study, and target population [107,108]. In athletes, vitamin D supplementation has shown conflicting results with measurements of athletic performance. While some studies reported an increase in maximal strength performance for leg extensions, leg curls, and chin ups [109,110], other studies found no change in strength when compared to a control [111,112,113,114,115]. Close et al. demonstrated an increase in anaerobic performance (vertical jump height and 10 m sprint) after 6 weeks of 5000 IU vitamin D supplementation [112], while other studies showed no effect on anaerobic performance after supplementation [111,113,115]. One study reported an increase in VO_2_ max after 5000 IU·d^−1^ for 8 weeks [114], while another reported no changes in VO_2_ max after 12 weeks of 3000 IU·d^−1^ [115]. No studies in athletes examined the effects of vitamin D supplementation on muscle mass.

In older adults, the effects of vitamin D supplementation on strength were overall positive. Hajj et al. reported a greater change in handgrip strength compared to a placebo (10.13 to 27.98 ng·mL^−1^, *p* < 0.001 versus 10.56 to 15.71 ng·mL^−1^, *p* < 0.001) [122]. Bauer et al. observed an increase of 0.79 kg in handgrip strength over 13 weeks (*p* = 0.005); however, this supplementation also included whey protein and leucine, which may have contributed to the increase in strength [118]. Multiple studies have examined the effects of supplementation on tests of muscle function including the Short Physical Performance Battery (SPPB), chair stand, time-up and go, postural sways, and tests of walking speed. Increases in SPPB and chair stand performances were observed after 800–1000 IU of vitamin D compared to a placebo [118,119], but no differences were reported in other studies [116,117,121,123,127]. Similarly, studies that examined the effects of vitamin D on muscle mass were generally positive. In those receiving vitamin D supplementation, appendicular muscle mass increased [118,122] and lean mass was maintained over 9 months while decreasing in the placebo group [119]. Additionally, Ceglia et al. reported a 10.6% increase in muscle fiber cross-sectional area and 29.7% increase in VDR concentration, indicating a potential mechanistic influence of vitamin D on skeletal muscle [82]. These results indicate that while vitamin D supplementation may have a potential positive influence on muscle strength and function in older adults, there is more evidence supporting a beneficial effect on muscle mass in this population. Additionally, in all populations reviewed, the dosage and duration of treatment varied, ensuing in inconclusive results.

Few studies were observed reporting the effects of vitamin D supplementation on skeletal muscle health in a youth population [124,125,126]. These studies also varied in age, duration, and dosage of vitamin D supplementation. While each intervention resulted in an increase in serum 25(OH)D levels, there were no positive effects in muscle mass, strength, or function when compared to a placebo [124,125,126]. This suggests that vitamin D supplementation may be more effective and necessary as an individual ages, potentially due to reduced dietary intake, reduced sun exposure and ability of the skin to produce vitamin D, and impaired absorption [128,129], as well as impairments in vitamin D actions on skeletal muscle through mitochondrial dysfunction and compromised anabolic pathways with age [130]. Several studies indicate a relationship between serum 25(OH)D levels and muscle mass and strength, suggesting a mechanistic link between low vitamin D levels and declines in muscle mass, strength, and function. Vitamin D deficiency has been extensively researched in sarcopenic older adults, suggesting that while deficiency of vitamin D may lead to sarcopenia and related adverse outcomes such as higher risk of falls, muscle fiber atrophy, and disability during hospitalization [131,132], supplementation has shown conflicting results in improving sarcopenia [133,134] However, the conflicting results in intervention studies cannot confirm vitamin D supplementation has an effective way to increase performance or prevent and/or treat sarcopenia in older adults. It is more likely that protein sources including a variety of nutrients including vitamin D have a greater effect. For example, 12–13-week interventions of supplementation including vitamin D and leucine-enriched whey protein showed improvements in muscle mass and lower extremity strength and function in sarcopenic older adults [8,118,135], specifically if adequate protein was consumed [8,118,135].

Although many prospective cross-sectional and intervention studies have examined vitamin D and skeletal muscle health (Table 1 and Table 2), conflicting results indicate that while there is potential for positive benefits of maintaining an adequate vitamin D status, there is no conclusive evidence that vitamin D levels beyond optimal range provide any enhancement to skeletal muscle health in a variety of populations. However, there appears to be a greater response in an older population, suggesting that maintaining levels through dietary intake and supplementation becomes more important with age. Additionally, the studies reviewed showed great diversity in participant characteristics, outcome measurements, and/or dosage amount and duration even within the separate population groups, which may have influenced the response or association between vitamin D and skeletal muscle health. 

### 3.2. Iron and Skeletal Muscle Health

#### 3.2.1. Iron and Skeletal Muscle Physiology

Iron is an essential mineral for multiple processes in the body that influence skeletal muscle performance such as oxygen transport, electron transport, and red blood cell production [136,137,138]. There is approximately 3–4 g of iron within the human body, in which about 70% of the body’s iron is found within hemoglobin (Hb) in red blood cells and myoglobin (Mb) within skeletal muscle [139]. Specifically, skeletal muscle contains about 10–15% of the iron in the body, mainly within oxidative fibers high in myoglobin [140]. The iron within the body is meticulously recycled to replace iron losses that occur within the gastrointestinal tract, skin, hair, sweat, and menses [141,142]. However, despite this efficient regulatory process, iron deficiency remains the most common nutritional deficiency in the world [139,143,144], typically from diminished iron absorption or increased iron loss, which is greater in females compared to males due to loss during menstruation [145]. Additionally, a vegan or vegetarian diet can be a risk factor for developing anemia, suggesting that dietary choices can be impactful for maintaining optimal iron status [146]. However, iron overload in thalassemia is an opposing challenge of iron deficiency that is influenced by iron regulation [147,148]. With the potential adverse health outcomes of iron overload, such as increased morbidity, maintaining an optimal iron status is necessary.

A number of biomarkers are utilized to reflect parts of the iron metabolism process, and therefore, are useful in isolation and in conjunction with one another. A common marker used to determine iron deficiency is ferritin. Ferritin is reflective of the body iron stores [149,150] and is used to determine the first stage of iron deficiency [139,151] defined as a lack of body iron stores. Ferritin levels <12–15 μg·L^−1^ indicate depleted iron stores; however, cutoff criteria between 15 and 35 μg·L^−1^ have often been utilized to diagnosis iron deficiency [47,152,153]. It is important to note that ferritin is an acute phase protein and is elevated with the presence of inflammation, so diagnosis with ferritin alone warrants caution in those with inflammation [154]. Correction for inflammation and measurement of inflammatory status are recommended when examining ferritin [155,156].

Decreased transferrin saturation or increased soluble transferrin receptor (sTfR) portrays the second stage of iron deficiency, reflecting reduced erythropoiesis. Transferrin saturation of <15–20% is considered indicative of iron deficiency [139]. An elevated sTfR indicates tissue iron deficiency and shows an inverse relationship with iron deficiency severity [157]. Together, the ratio of serum sTfR and serum ferritin can provide an index that reflects body iron. This measurement is effective for monitoring fluctuations in iron status [158].

The last stage of iron deficiency results in iron deficiency anemia with the addition of Hb as a biomarker [159]. Concentrations of Hb is a commonly measured parameter due to its affordability and accessibility; however, Hb is not specific to iron due to other potential contributors such as folate or vitamin B12 deficiency or anemia of chronic inflammation [139,160]. The inclusion of a second biomarker, such as ferritin or sTfR, simultaneously with Hb can confirm the diagnosis of iron deficiency anemia. Cutoff values range based on sex, age, and ethnicity [161]. According to the World Health Organization [159], diagnosis of anemia utilizing Hb concentrations have cutoff criteria of <115 g·L^−1^ for ages 5 to 11 years, <120 g·L^−1^ for ages 12–14 years, and <120 g·L^−1^ and 130 g·L^−1^ for females and males, respectively, 15 years or older [159,162].

Iron is essential for skeletal muscle function largely due to several pathways. While known for its necessary role for Hb production in red blood cells, iron is required for many processes for energy metabolism [163,164]. In particular, oxidative metabolism requires iron for adequate oxygen supply and the transfer of electrons during redox reactions [165,166]. Additionally, the majority of iron in skeletal muscle is within slow twitch muscle fibers that are abundant in myoglobin, in which oxidative metabolism occurs [166,167]. Enzymatic complexes within the electron transport chain rely on iron to function, indicating that an adequate supply of iron is essential for the oxidation of fuel sources for energy [164,165]. This indicates that the aerobic capacity of an individual greatly relies on the oxygen-carrying capacity of the blood as well as the muscle oxidative capacity [140], both of which are heavily dependent upon iron.

#### 3.2.2. Iron and Skeletal Muscle Health

Multiple studies have examined associations between iron status and parameters of skeletal muscle health (Table 3). In youth populations, the association between iron biomarkers and performance seemed to largely depend on what performance metric were utilized. Wang et al. reported that iron deficiency was related to lower fat-free associated VO_2_max within females, and both males and females with iron deficiencies had lower energy expenditure at leisure compared to adequate iron group [168]. Arsenault et al. reported that females with low ferritin levels had lower levels of performance during a shuttle run test, whereas males with low ferritin levels had lower long jump scores compared to those of normal ferritin levels [169]. Lastly, in a study conducted by Gracia-Marco et al. Hb concentrations were associated with estimated VO_2_max results in male adolescents only [99]. These varying outcomes provide insight into iron deficiency playing a role in oxygen transport directly influencing aerobic activities. The influence of iron status on anaerobic activities is further warranted to understand the role of iron on muscle strength, health, and function within youth populations.

Similar observations were observed in athletic populations where iron status seems to be more influential to the performance of aerobic-based activities. In a study conducted by DellaValle & Hass, female rower athletes that were categorized as iron depleted without anemia (IDNA) (serum ferritin < 20.0 μg·L^−1^), had lower VO_2_max and higher blood lactate concentrations during a 4 km rowing test [46]. In addition, the authors suggested that iron status also likely played a role in training load of the athletes where those athletes categorized as IDNA had lower training times than the non-anemic group during a 4-week observation. In addition, Tsai et al. reported that mildly anemic males enlisted within the Taiwan Military were likely to be the worst 10% performers during a standard 3000 m run test but were likely to be the best 10% performers during an anaerobic test such as the 2 min push-up test [170]. Shoemaker et al. also indicated within youth athletic population that performance during aerobic fitness tests such as vertical jump, broad jump, agility drill times, 20-yard dash time, power push up force, were related to Hb status in males and with sTfR and iron intake in females [171]. Together these studies further support the notion that iron plays a role in aerobic metabolism and cardiorespiratory fitness; however, the role on iron status and anaerobic fitness tests require further investigation.

Within the older adult population, iron status seems to play a role in frailty. For example, multiple studies reported that lower Hb concentrations were associated with higher frailty scores or associated with a greater risk of frailty than non-frail individuals [172,174,176,177]. However, in older adults, iron status seemed to vary based on muscle strength metrics. For example, low Hb levels were reported to not be associated with grip strength in older adults in Brazil [174]; however, in hospitalized older adults there was a positive association between Hb levels at baseline and at discharge within those with iron deficiency. Together, these associations suggest that low iron status is related to decreased aerobic and anaerobic performance. While few studies examined the associations between iron status and muscle mass, low Hb concentrations were related to lower muscle mass and the presence of frailty in older adults [172,173,174,175,176]. However, there was no concluding evidence on muscle strength and function measurements.

#### 3.2.3. Iron Interventions and Skeletal Muscle Health

Iron supplementation is a common method utilized to correct iron deficiency, particularly in athletic populations. Oral supplementation doses ranging from 40 to 400 mg·d^−1^ for treatment durations of 6–24 weeks has been effective in improving iron status [178,179,180,181]. However, there are contrasting results regarding the effectiveness of iron supplementation for performance measurements reflective of skeletal muscle health. Multiple studies have examined the effects of iron supplementation on performance in athletes, yet there is a lack of studies examining if iron supplementation influences skeletal muscle mass or is effective in other healthy populations such as youth or older adults (Table 4). In aerobic-based athletes (rowers, runners, and cyclists), iron supplementation over 6 weeks was effective in improving aerobic performance such as 4 km time trial and VO_2_max for rowers and runners [179,180]. However, a shorter intervention of 80 mg·d^−1^ showed no improvement in muscle recovery from cycling performance [182]. One study examined muscle strength measurements in female volleyball players. Iron supplementation of 325 mg·d^−1^ over 11 weeks improved strength in two power exercises and total strength over six different exercises [181]. These studies indicate that improving iron status via supplementation may be effective in improving performance-based measurements of skeletal muscle health. Future research should examine the effects of improving iron status on skeletal muscle mass, strength, and function in other vulnerable populations such as youth and older adults.

### 3.3. Interrelationship between Vitamin D, Iron, and Chronic Inflammation

#### 3.3.1. Anti-Inflammatory Role of Vitamin D

In addition to its role on skeletal muscle health, vitamin D is thought to have anti-inflammatory actions through activation and differentiation of inflammatory cells, leading to a reduction in risk of infection and inflammation [12,13,183,184]. Vitamin D has immunomodulatory benefits including the enhancement of antimicrobial peptides and defensins to improve cellular immunity and reduce cytokine storms linked to infection. Adequate levels of vitamin D are related to the decrease in production of pro-inflammatory cytokines and an increase in anti-inflammatory cytokines [14,15,16,17,18]. Additionally, Shoemaker et al. recently reviewed the effects of vitamin D supplementation on reducing the risks of respiratory tract infections and viral infections including SARS-CoV-2 and indicate that sub-optimal vitamin D status increases risk for incidence, complication, and mortality due to infection and the presence of inflammation (manuscript in press). Therefore, vitamin D is a potential nutritional strategy that may reduce chronic inflammation.

The relationship between vitamin D status and inflammation has been studied previously in older adults [185,186]. Furthermore, vitamin D supplementation has demonstrated beneficial effects on chronic inflammation [7,14,15,16,187]. For example, Liberman et al. reported that 13 weeks of vitamin D and protein supplementation was effective in preventing increases in inflammatory cytokines compared to a placebo in older adults [7]. Similarly, Pereira et al. reported that 12 weeks of oral nutritional supplementation rich in vitamin D, HMB, and protein improved multiple biomarkers related to inflammation, immune function, and overall muscle health [188].

#### 3.3.2. Connection between Vitamin D and Iron Status

**Figure 1:**
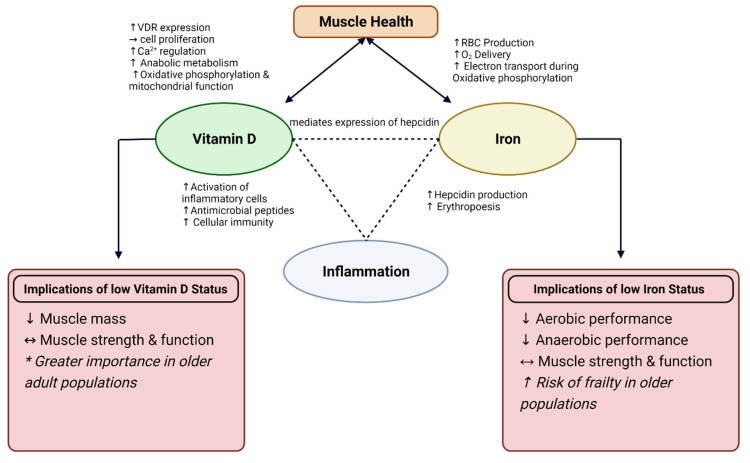
Interaction between Vitamin D and Iron on muscle health and role for inflammation. Depicted implications of low Vitamin D and Iron status on muscle health and performance. VDR = Vitamin D Receptor; RBC = red blood cell, Ca^2+^ = calcium; O_2_ = oxygen; ↑ = increase; ↓ = decrease; ↔ = conflicting results. * More profound results observed in interventions with older adults.

Vitamin D and iron are both essential nutrients for skeletal muscle health, suggesting that optimal status in both micronutrients may interactively benefit skeletal muscle health. Vitamin D is important for the regulation of iron metabolism; therefore, low vitamin D status may result in low iron status [9,11,34]. Due to the relationship observed between vitamin D action on pro-inflammatory cytokines and mechanisms behind iron regulation [9,35], examining the physiological functions of vitamin D and iron status on skeletal muscle health and inflammation is an important next step in promoting health and performance. This theorized interaction is displayed in Figure 1. The connection between vitamin D and iron status is thought to be associated with hepcidin, an antimicrobial peptide that is essential for the regulation of iron metabolism [9,35]. Iron absorption and excretion is a highly regulated process, in which iron absorption increases with deficiency and decreases when iron stores are full. Systemic iron status, erythropoiesis requirements, and presence of inflammation can all influence this regulatory process [136]. The presence of high inflammation results in an increase in hepcidin production, causing iron to sequester and limit iron-supported erythropoiesis [189]. This leads to diminished ability to absorb iron, thus leading to iron deficiency anemia.

Additionally, vitamin D can mediate the expression of hepcidin through the binding of VDR with a gene promotor called HAMP gene to downregulate hepcidin production [35]. Furthermore, the role vitamin D has in decreasing expression of inflammatory cytokines that have a stimulating role on hepcidin production may indirectly contribute to this integration. Through in vitro studies, there is evidence that adequate levels of vitamin D are associated with reduced concentrations of hepcidin due to the suppression of the HAMP gene, as well as due to reduced concentrations of pro-inflammatory cytokines such as IL-1β and IL-6 [11]. This suggests the potential for vitamin D levels to influence iron regulation through hepcidin, specifically in the presence of inflammation. Additionally, vitamin D supplementation has been found to decrease hepcidin, and thus may have benefits in altered iron status, particularly in those with chronic inflammation [11]. While typically associated with anemia related to inflammation, it is possible that this mechanism may also be related to iron deficiency with or without anemia due to the reduction in iron necessary to support erythropoiesis. This suggests that those with chronic inflammation may have greater iron requirements to increase circulating iron concentrations and promote red blood cell production, indicating the need for nutritional support with both vitamin D and iron.

Relationships between low vitamin D status and low iron status are reported, providing further support for this nutritional interaction [49,50,190]. Vitamin D deficiency and low iron status are prevalent in multiple populations including older adults [22,40,44,45,51], children [52,53,54,55], and athletes [47,56,57], emphasizing the potential influence these nutrients may have on skeletal muscle health throughout the lifespan. Additionally, associations between vitamin D deficiency and low iron status have been demonstrated. For example, Malczewska-Lenczowska et al. reported that female athletes with iron deficiency also had lower vitamin D concentrations. Female athletes with vitamin D deficiency also had lower ferritin and iron concentrations and higher total iron binding capacity and sTfR, indicating low iron status [49]. Additionally, vitamin D supplementation (3000 IU·day^−1^) was effective in preventing a decline in Hb and hematocrit and improve transferrin levels, as well as concentrations of vitamin D in elite male rowers [191]. These findings support the association between vitamin D and iron status in athletes, although it is unclear which of the nutrients is the cause or the effect in the relationship. Further research is needed to examine if this nutrient interaction is influential to skeletal muscle health.

A retrospective study in children aged 10–20 years demonstrated an association between vitamin D deficiency and both anemia and iron deficiency when accounting for contributing factors [192]. The relationship between Hb and vitamin D was more prominent in female children, compared to males, suggesting that those vulnerable to nutritional deficiencies may be most affected by this nutritional interaction with skeletal muscle health through the growth and development stage. Similarly, in a pediatric population of inflammatory bowel disease patients, children with vitamin D concentrations ≥ 30 ng mL^−1^ had lower hepcidin and higher Hb concentrations when controlling for inflammation [193]. In older adults, the prevalence of vitamin D deficiency was higher in those with anemia due to inflammation (56%) and nutritional deficiency (48%) [51]. These findings suggest that children and older adults are at risk for compounding nutritional deficiencies, along with inflammation, that may be influential to the muscle growth and atrophy typically observed at each life stage. Therefore, adequate consumption of nutrients such as iron and vitamin D is essential for these populations.

### 3.4. Animal Food Sources

Adequate consumption of vitamin D and iron may be key in enhancing muscle mass, strength, and performance. Animal-source foods are abundant in vitamin D and iron, which may help individuals reach optimal, bioavailable intakes of these nutrients to support skeletal muscle health [58,59,60]. Specifically, beef is rich in bioavailable heme iron that may reduce the risk of iron deficiency and anemia [194,195]. Heme iron is found only in animal-source foods and are better absorbed than plant sources containing non-heme iron [195]. In particular, beef sources including ground beef, beef liver, and bottom round beef cuts are abundant sources of iron, containing 2–5 g of heme iron per 3 oz. serving. Previous studies have indicated the consumption of iron-rich red meat, along with resistance training, have shown beneficial effects on muscle mass, muscle strength, and reduce inflammatory markers [58,59], providing support for iron’s role in muscle health.

While vitamin D originates from sunlight exposure, dietary intake of vitamin D can be obtained from a variety of food sources in which approximately 60% of intake comes from animal-source foods such as fish, meat, and eggs [196]. Dietary intake of vitamin D range from 3.8 to 7.2 µg·d^−1^ in youth, 3.6 to 5.4 µg·d^−1^ in adults, and 3.9 to 5.1 µg·d^−1^ in older adults [197], which is lower than the recommended intake between 10 and 15 µg·d^−1^ (400–600 IU) [198]. Fortified milk provides a majority of the vitamin D within the American diet [199], with approximately 3 µg per cup [200]. Additionally, other key sources of vitamin D include beef liver and other beef sources, fatty fish such as salmon, eggs, and chicken [201]. Previous studies have observed positive results in vitamin D status after supplementing with vitamin D-fortified milk [202], suggesting potential for increasing muscle health through food sources.

Adequate nutritional intake is essential for muscle growth, performance, and preventing of sarcopenia. In particular, nutrients abundant in animal food sources such as vitamin D, iron, and protein, have been related to athletic performance, functional performance, and muscle growth [58,59,203,204,205]. Criticism of the current Recommended Daily Allowance (RDA) of 0.8 g·kg^−1^·d^−1^ warrants an increase specifically when protein anabolism is effected such as during the aging process and during exercise [206,207]. Sarcopenic older adults had lower intakes of protein, lipids, and micronutrients including iron and vitamin D [208,209]. Additionally, oral nutritional supplementation rich in protein and vitamin D resulted in improvement of markers of health, strength, and inflammation in malnourished, sarcopenic older adults [188]. These results indicate that an animal-source food matrix may be optimal when trying to enhance skeletal muscle function and reduce the risk of chronic inflammation.

Dietary protein from animal sources has long been established as beneficial for skeletal muscle by increasing muscle protein synthesis due to the essential amino acid content [210,211]. Animal protein sources effectively improved skeletal muscle strength and mass in healthy young adults and older adults [58,59,203,212,213], indicating functional benefits of including dietary animal-source foods. Red meat, such as beef, and dairy products are two groups of animal-source foods that show promise for improving skeletal muscle health, potentially due to the nutrient content of these foods.

Multiple studies have examined the effects of beef intake on skeletal muscle-based outcomes in older adults [58,59,214,215,216]. Recent reviews have concluded that beef and/or the nutrients found within beef may improve muscle function [214,217]. Asp et al. investigated the relationship between beef intake and muscle mass in older adults ages 60–88 years, reporting that beef intake was positively related to mid-arm muscle area. Furthermore, regression analysis predicted that a 1 oz increase in beef consumption per day would result in a 2.23 cm^2^ increase in mid-arm muscle area [58]. In agreement, Morris & Jacques also predicted a linear increase in muscle mass (appendicular skeletal muscle index) in association with a 100 g per week increase in beef intake [213]. Lean red meat enhanced the effects of resistance training on muscle mass and strength in older females [59] and in 1 repetition maximum leg extension strength in older males [215]. In contrast, when examining the effects of lean beef in addition to resistance training, no additional benefits were observed in older adults when beef was consumed twice a week for 24 weeks [216]. Additionally, lean beef and protein supplementation had no positive effect on fat-free mass in older adults [215,218]. Meals containing pork, beef, or chicken showed similar impact on body composition and strength, indicating that high-quality protein sources, in general, have the same effect on skeletal muscle health [219,220]. Furthermore, consumption of beef protein isolate, chicken protein isolate, or whey protein all resulted in increases in lean body mass, regardless of source of protein [203]. Higher intake of protein source foods including red meat, poultry, fish, dairy, soy, nuts, seeds, and legumes were positively associated with higher percent skeletal muscle mass over time in older adults, indicating that higher intake of animal-source foods can help maintain skeletal muscle with age [212]. These studies indicate conflicting skeletal muscle health outcomes with animal protein consumption; however, adequate consumption of protein sources in general appear to be a beneficial method for preserving muscle mass and strength. Future random controlled trials are required to provide further support of animal-source foods such as beef and poultry as dietary sources to promote skeletal muscle health.

Fortified dairy products are also nutrient-dense foods with the potential to improve skeletal muscle health [204,205]. In addition to many dairy products being fortified with vitamin D, they are rich in protein and many micronutrients including vitamin B12, calcium, riboflavin, and zinc, that are important for muscle health and function [221]. Consumption of dairy milk with additional protein improved fat-free mass, strength, and power in young males when consumed following resistance training [222]. However, in older adults, consuming higher protein dairy milk did not further improve the benefits obtained from resistance training alone on fat-free mass, power, or physical performance, but did improve maximal strength measurements [223].

## 4. Conclusions

Vitamin D and iron are nutrients that have a role in enhancing skeletal muscle health. While the supplementation of these nutrients has conflicting results, overall, vitamin D in combination with protein has been shown to increase muscle mass, as well as high-protein foods rich in vitamin D and iron. There was an overall positive effect of vitamin D on muscle mass, with mixed results on muscle strength and function. The influence of vitamin D appeared to be more profound within older adults. There was an overall positive effect of iron on both aerobic and anaerobic performance, with mixed results on muscle strength and function. While vitamin D and iron deficiency commonly occur simultaneously, there are few studies examining the effects this has on skeletal muscle health and inflammation. Future studies are required to examine if adequate dietary intake of these nutrients influence levels of inflammation.

While consumption of isolated nutrients such as iron and vitamin D may have some positive outcomes, an interaction of combined nutrients, in addition to physical activity, is most effective in improving skeletal muscle health, thus promoting qualities that will reduce inflammation and promote health well-being. Due to the high nutrient density, animal-source foods, including animal protein sources such as lean beef and dairy products, can provide a nutrient matrix that may be necessary for optimal absorption and utilization to promote skeletal muscle health. Thus, there is a need for future trials examining the role animal-source foods and associated nutrients have on skeletal muscle health in populations that may have higher nutritional requirements such as older adults, youth, and athletes.

## Figures and Tables

**Table 1:** Cross-sectional studies examining associations between vitamin D status and muscle health.

Author, Year, Country	Study Participants (Mean ± SD)	Measurements	Conclusions
Athletes			
Koundourakis et al., 2014, Greece [84]	Caucasian male soccer players, mean age: 25.6 ± 6.2 years *n* = 67	Serum 25(OH)D concentrations and performance of the squat jump, countermovement jump, sprint performance, and VO_2_max,	Concentrations of serum 25(OH)D were positively associated with an increase in performance during the squat jump, counter movement jump, VO_2_max (r = 0.394–0.740), and negatively associated with sprint performance at 10 m and 20 m (r = −410–−0.649) before the soccer season (pre) and during the six-week off-season period (post) (*p* > 0.01)
Fitzgerald et al., 2014, United States [89]	Professional male ice hockey players, mean age: 20.1 ± 1.5 *n* = 52	Serum 25(OH)D concentrations and performance during skate treadmill graded exercise testing	Concentration of serum 25(OH)D was not associated with VO_2_max, max heart rate, peak respiratory exchange ratio, final stage completed, and total exercise time completed during the graded exercise test (*p* = 0.22–0.71)
Hamilton et al., 2014, Qatar [85]	Male soccer players stratified based on 25(OH)D concentration (<10–>30 ng/mL) mean age: 24.4 ± 8.1 *n* = 342	Serum 25(OH)D concentrations and lower limb isokinetic performance (peak torque)	Soccer players with serum 25(OH)D levels >30 ng·mL^−1^ displayed 17% greater concentric and 13% greater eccentric hamstring peak torque in the non-dominant leg compared to those with 25(OH)D levels of ≤10 ng·mL^−1^ (*p* = 0.015–0.021)
Forney et al., 2014, United States [90]	Recreationally active college students, mean age: 23.0 ± 0.7 years *n* = 39; *n* = 20 males, *n* = 19 females	Serum 25(OH)D concentrations and performance during aerobic testing (Bruce Protocol [VO_2_max]), anaerobic power (Wingate), strength (upright bench press, bicep curl, triceps pushdown, leg curl, leg extension, and upright row [8-repetition max]), and power (maximal vertical and horizontal jump).	Concentrations of serum 25(OH)D were associated with VO_2_max (r = 0.360, *p* = 0.018), however, there was no association of 25(OH)D with anaerobic power, muscular strength, and muscular power
Ksiazek et al., 2016, Poland [86]	Polish premier league soccer players, mean age: 22.7 ± 5.3 years *n* = 43	Serum 25(OH)D concentrations and performance during hand grip strength, lower-limb isokinetic strength, and aerobic performance (VO_2_max)	Soccer players with serum 25(OH)D concentrations >20 ng·mL^−1^ displayed a 12% greaterpeak torque compared to those with 25(OH)D levels of ≤20 ng·mL^−1^ (*p* ≤ 0.05) A significant positive correlation between 25(OH)D levels and concentric leg extension peak torque (r = 0.410, *p* < 0.040).
Zeitler et al., 2018, Austria [87]	Healthy recreational athletes age: 18–65 years; 40.5 ± 9.2 (males), 38.7 ± 9.8 (females) *n* = 581; *n* = 287 males, *n* = 284 females	Serum 25(OH)D concentrations and performance during maximal and submaximal treadmill running	Males with serum 25(OH)D levels <20 ng·mL^−1^ had significantly lower submaximal performance on the treadmill compared with those with normal 25(OH)D levels (*p* = 0.045) Associations between 25(OH)D levels and maximal and submaximal treadmill performance in males and females displayed no significant differences
Most et al., 2021, Germany [88]	88 male handball and 24 male ice hockey players stratified based on 25(OH)D concentration (<30 and >30 ng/mL) mean age: 26.1 ± 5.2 years *n* = 112	Serum 25(OH)D concentrations and performance during a maximal cycle ergometer test (W/kg)	Athletes with serum 25(OH)D levels <30 ng·mL^−1^ achieved an 11% higher maximal aerobic power compared to those with insufficient levels (>30 ng·mL^−1^) (*p* = 0.030)
Older Adults			
Marantes et al., 2011, United States [91]	Age-stratified, random sample of males and females ages 21–97 years old, mean age: 57.0 ± 18.0 years. *n* = 700; *n* = 325 males, *n* = 375 females	Concentrations of serum 25(OH)D and 1,25(OH)_2_D, fat mass and muscle mass, handgrip strength, isometric leg extension strength	Lower serum 25(OH)D levels were inversely associated with greater fat mass, while lower 1,25(OH)_2_D levels were positively associated with lower muscle mass and muscle strength in males and females
Mastaglia et al., 2011, Argentina [4]	Females over age 70 years attending bone health assessments at the Buenos Aires Hospital, mean age: 71.0 ± 4.0 years. *n* = 54	Lower limb lean mass, muscle function (walking speed, chair stand, balance), muscle strength (hip flexors and abductors, leg extensors), levels of calcium, phosphorus, serum 25(OH)D concentrations, and urinary calcium and creatinine	Older adults with serum 25(OH)D levels ≥20 ng·mL^−1^ (*n* = 25) had 11% better scores on muscle function tests, 0.4 s faster walking speed, and were 14% and 13% stronger in tests of leg extension and hip abduction strength, respectively, than those with serum 25(OH)D levels <20 ng·mL^−1^ (*n* = 29).
Toffanello et al., 2012, Italy [20]	Older adults aged 65–98 years from a large cohort study in Italy (Pro.V.A), mean age: 75.6 ± 7.5. *n* = 2694	Physical Performance (balance, chair stand, gait speed, 6 min walking test), handgrip strength, quadriceps strength, levels of PTH andserum 25(OH)D	Levels of serum 25(OH)D were positively associated with the chair stand, gait speed, 6 min walking test, and handgrip strength (*p* < 0.001). Concentrations of 100 nmol·L^−1^ was determined to be related to greater muscle function
Tieland et al., 2013, The Netherlands [92]	Older adults >age 65 years who were considered frail or pre-frail, mean age: 79.0 ± 7.8 years. *n* = 127	Serum 25(OH)D, creatinine, glucose, and insulin concentrations, dietary intake, body composition, leg strength (leg press and leg extension), handgrip strength, and physical performance (SPPB)	Levels of serum 25(OH)D were associated with appendicular skeletal muscle mass (β = 0.012, *p =* 0.050). Levels of 25(OH)D and vitamin D intake were positively associated with higher SPPB scores (β = 0.020–0.180, *p* = 0.020–0.038).
Gumieriro et al., 2015, Brazil [93]	Older adults with a hip fracture and older than 65 years admitted to hospital, mean age: 80.0 ± 7.0 years. *n* = 100	Serum 25(OH)D concentrations, handgrip strength, mid-upper arm muscle circumference, length of hospital stay, mortality	Participants with lower serum 25(OH)D concentrations had 40% lower handgrip strength and 52% higher mortality rate. Levels of serum 25(OH)D predicted handgrip strength when adjusted for age and sex (β = −1.945, *p* = 0.020).
Iolascon et al., 2015, Italy [94]	Post-menopausal females aged 50 years or older, mean age: 65.9 ± 7.7 years. *n* = 80; *n* = 46 with low vitamin D levels, *n* = 34 with normal vitamin D levels	Handgrip strength, isometric leg extension strength, SPPB, gait speed, serum 25(OH)D concentrations	Serum 25(OH)D concentrations were positively associated with handgrip strength (r = 0.234), leg extensor strength (r = 0.234), and inverselyl associated with time to complete physical performance tests such as walking speed (r = −0.457) and chair stand (r = −0.564). Those with serum 25(OH)D levels ≥30 ng·mL^−1^ had better results for handgrip strength, leg extension strength, and SPPB scores (*p =* 0.001–0.003)
Verlaan et al., 2017, The Netherlands [6]	Subsample of sarcopenic participants from the PROVIDE study, which were ≥65 years old, mean age: 71.0 ± 4.0 years. *n* = 132; sarcopenic participants (*n* = 66) and non-sarcopenic controls (*n* = 66)	Body composition (appendicular muscle mass and fat mass), muscle strength and function (handgrip strength, SPPB), ADLs, frailty status, nutritional status, and levels of serum 25(OH)D, vitamin B12, and folate	Serum 25(OH)D levels were not different between groups; however, there was a greater prevalence of vitamin B12 deficiency in sarcopenic individuals
Aspell et al., 2019, Ireland [95]	Older adults aged 60 years or older from the English Longitudinal Study of Aging, mean age: 69.8 ± 6.9 years. *n* = 4157	Serum 25(OH)D concentrations, handgrip strength, SPPB	A greater number of older adults had low handgrip strength and SPPB score in the lowest serum 25(OH)D concentration quintile compared to the others quintiles (*p* < 0.0001–0.01). After adjusting for confounding factors, vitamin D deficiency was positively associated with low SPPB score [OR 1.65, *p* < 0.01) and positively predicted low handgrip strength (OR 1.44, *p* < 0.001).
Conzade et al., 2019, Germany [96]	Older adults aged 65 years or older, mean age: 75.7 ± 6.6 years. *n* = 702	Muscle mass, handgrip strength, gait speed, TUG, Serum 25(OH)D levels	Low levels of serum 25(OH)D (<25 nmol·L^−1^) were had a 0.94% greater loss in muscle mass and 3.06% increase in time to complete TUG compared to higher levels (≥50 nmol·L^−1^) but was not related to change in handgrip strength or gait speed.
Vaes et al., 2019, The Netherlands [97]	Older adults 65 years or older that attended the screening visit of two clinical trials (D-DOSE and D-FIT), mean age: 74.0 ± 6.0 years. *n* = 756	Serum 25(OH)D levels, handgrip strength, gait speed, TUG, isometric leg extension strength	Older adults with lower serum 25(OH)D levels (<50 nmol·L^−1^ and 50–75 nmol·L^−1^) had inverse relationships for time to complete TUG (β = 0.73–0.83, *p =* 0.01–0.05) and lower scores for gait speed (β = −0.04, *p* < 0.05), but there no relationships observed with handgrip or leg extension strength. Those with lower 25(OH)D levels were also more likely to be categorized as frail.
Youth			
Dong et al., 2010, United States [98]	Adolescents aged 14–18 years old, mean age: 16.2 ± 1.2 years. *n* = 599	Levels of serum 25(OH)D, time spent in physical activity, cardiovascular fitness determined from oxygen consumption during a treadmill test	Positive associations were found between serum 25(OH)D levels and unadjusted and adjusted vigorous physical activity (r = 0.132–0.139, *p =* 0.002–0.01) and maximal oxygen consumption (r = 0.100–0.212, *p* < 0.01–0.025).
Gracia-Marco et al., 2012, Europe (Sweden, Greece, Italy, Spain, Hungary, Belgium, France, Germany, Austria) [99]	Adolescents aged 12.5–17.5 years old across Europe that completed the blood sample analysis as part of the HELENA-CSS study, mean age: 15.0 ± 1.2 years. *n* = 1089; males, *n* = 509, females, *n* = 580	Standing long jump, 20 m shuttle run to estimate VO_2_max, red blood cell parameters, biomarkers of iron status (sTfR and ferritin), other micronutrients (vitamins A, E, C, B6, and B12, folate, and serum 25(OH)D) concentrations	Concentrations of serum25(OH)D were positively correlated with estimated VO_2_max (from 20 m shuttle run) (β = 0.091, *p* = 0.030) and standing broad jump (β = 0.125, *p* = 0.010) in female adolescents.
Valtueña et al., 2013, Europe (Sweden, Greece, Italy, Spain, Hungary, Belgium, France, Germany, Austria) [100]	European adolescents ages 12.5–17.5 years, mean age: 14.9 ± 1.2 years. *n* = 3000, *n* = 1006 had samples for 25(OH)D and included in the analysis	Serum 25(OH)D concentrtions, BMI, fat mass, fat-free mass, fat mass index, fat-free mass index, 20 m shuttle run to estimate VO_2_max, handgrip strength, standing long jump	In males, VO_2_max had a positive correlation with serum 25(OH)D concentrations (r = 0.108, *p* = 0.022). Linear regression demonstrated a positive association between VO_2_max and serum 25(OH)D concentrations (β = 0.189, *p =* 0.002) and a negative associaton between BMI and serum 25(OH)D concentrations (β = −0.125, *p =* 0.023). In females, handgrip strength was positively associated with serum 25(OH)D concentrations (β = 0.168, *p =* 0.002). Greater long jump performance was a positively ssociated with higher serum 25(OH)D levels in males.
Carson et al., 2015, Ireland [101]	Males and females ages 12 and 15 years from Northern Ireland. *n* = 1015; (12-year-old males, *n* = 266; 12-year-old females, *n* = 260; 15-year-old males, *n* = 239; 15-year-old girls, *n* = 250)	Serum 25(OH)D concentrations, BMI, fat mass, fat-free mass, fat-free mass index, handgrip strength, jump height, jump power, 20 m shuttle run to estimate VO_2_max	Serum 25(OH)D concentrations in the highest tertile (>51 nmol·L^−1^) were positively associated with greater muscle strength in the 15-year-old males (β = 3.90, *p* < 0.001), but this relationship was not present in any other group categorized by age or sex. There were no associations between serum 25(OH)D concentrations and muscle mass, muscle power or VO_2_max
Bezrati et al., 2016, Tunisia [102]	Physically active males aged 7–15 years, mean age: 11.4 ± 2.0, 11.8 ± 2.2, and 11.0 ± 1.9 years for vitamin D deficient, insufficient, and sufficient, respectively. *n* = 125	Serum 25(OH)D concentrations, body fat percentage, vertical jump, broad jump, triple hop, sprint agility, and trunk force	Serum 25(OH)D levels were positively associated with trunk force, vertical jump, and broad jump (β = 0.165–0.552, *p* < 0.001) and inversely related to 10 m sprint, 20 m sprint, and shuttle run (β = −4.330–06.436, *p <* 0.001).
Blakeley et al., 2018, United States [103]	Children in fourth through eighth grades in Boston area, mean age: 11.2 ± 1.3 years. *n* = 350	Handgrip strength, levels of HDL cholesterol, triglycerides, and serum 25(OH)D concentrations, BMI	There were no associations between handgrip strength and serum 25(OH)D concentrations.
Wakayo et al., 2018, Ethiopia [104]	Ethiopian school-age children 11–18 years old, median age: 15 years. *n* = 174	Serum 25(OH)D concentrations, handgrip strength	There was no association between handgrip strength and serum 25(OH)D levels

**Table 2:** Experimental studies examining the effects of vitamin D supplementation on muscle health.

Author, Year, Country	Study Participants	Supplemental Treatment	Duration	Measurements	Conclusions
Athletes					
Shanely et al., 2014, USA [111]	Professional football, tennis, lacrosse, baseball players, and professional wrestlers. *n* = 33 mean age: 16.3 ± 0.25 years.	600 IU·d^−1^ (Portobello mushroom powder) vs. Placebo	6-weeks	Serum 25(OH)D concentrations, isometric deadlift strength and vertical jump performance	No associations between serum 25(OH)D concentrations with isometric muscle strength or vertical jump performance. Isometric strength and vertical jump performance was not different between supplement and placebo group.
Close et al., 2013, UK [112]	Professional rugby, soccer, flat jockeys and national hunt jockeys. *n* = 61	5000 IU·month^−1^ vs. placebo	6-weeks	Serum 25(OH)D concentrations, isometric strength, 10 m sprint performance and vertical jump performance	There was approximately a 3-inch increase in vertical jump height (*p =* 0.008) and 0.04-s time improvement in 10 m sprint performance (*p* = 0.008) in the supplementation group with no change in the placebo.
Close et al., 2013, UK [113]	Professional rugby and soccer players. *n* = 30	20,000 or 40,000 IU·week^−1^ vs. placebo	6 or 12-weeks	Serum 25(OH)D concentrations, dynamic strength (1-RM bench press, 1-RM leg press) and vertical jump performance	Serum 25(OH)D concentrations increased in both 6-week and 12 week periods (*p <* 0.0005) with concentrations higher after 6-weeks of 40,000 IUs compared to 20,000 IUs (*p* = 0.016). However, serum 25(OH)D concentrationswere not associated with improvements in 1-RM bench press, 1-RM leg press and vertical jump performance following 6 or 12-week of supplementation.
Jastzebska et al., 2016, Poland [114]	Well trained soccer players. *n* = 36	5000 IU·d^−1^ vs. placebo	8-weeks	Serum 25(OH)D concentrations, 30-s Wingate test for peak power, sprint tests for 5, 10, 20, and 30 m, squat jump, countermovement jump	Supplementation group displayed an increase in all power tests except for 30 m sprint time (*p* < 0.001); however, mean change scores were not different between supplementation and placebo groups.
Todd et al., 2016, Ireland [115]	Gaelic football players. *n* = 42	3000 IU·d^−1^ vs. placebo	12-weeks	Serum 25(OH)D concentrations and VO_2_max	Serum 25(OH)D concentrations increased following supplementation, however, supplementation had no effect on VO_2_max.
Wyon et al., 2016, UK [109]	Judo athletes. *n* = 22	150,000 IU once vs. placebo	8 days	Serum 25(OH)D concentrations, maximal isokinetic leg extension and leg curls	Supplement group displayed a 13% increase in muscle strength following 8 days of supplementation (*p* ≤ 0.001).
Fairbairn et al., 2017, New Zealand [110]	Professional rugby players. *n* = 57	50,000 IU once every 2 weeks	11–12 weeks	Serum 25(OH)D concentrations, 30 m sprint performance and maximal dynamic strength (weighted chin-up 1-RM, bench pull 1-RM, and bench press 1-RM)	No difference in 30 m sprint performance; however, there was a 5.5 kg increase in dynamic strength (weighted chin-up 1-RM), (*p* = 0.002).
* Lips et al., 2010, USA, Mexico, The Netherlands, Germany, Canada [116]	Older adults aged 70 years or older who were ambulatory and had 25(OH)D levels between 6 and 20 ng·ml^−1^, mean age: 77.6 ± 6.6 and 78.5 ± 62.0 years in the placebo and experimental groups, respectively. *n* = 226 randomized, 202 completed the study.	8400 IU vitamin D_3_ weekly (*n* = 105) or placebo (*n* = 97)	16 weeks	Serum 25(OH)D concentrations, postural sway, SPPB, and levels PTH	25(OH)D levels increased from approximately 14 to 26 ng·mL^−1^ (*p* < 0.001) in the supplementation group; however, there were no changes in postural sway or SPPB scores for either group
** Ceglia et al., 2013, USA [82]	Older females aged 65 years or older who were ambulatory, community-dwelling, and postmenopausal, mean age: 78.0 ± 5.0 years. *n* = 24 randomized, 21 included in analysis	4000 IU vitamin D_3_ (*n* = 11) or placebo (*n* = 13)	4 months	Serum 25(OH)D concentrations, leg extension strength, muscle fiber type and intramyonuclear VDR from biopsies of the vastus lateralis	The supplementation group had a much greater increase in 25(OH)D levels after 4 months (36.4 vs. 4.2 nmol·L^−1^ increase, *p <* 0.001), as well as a 10.6% increase in total muscle fiber cross-sectional area (*p =* 0.048) and 29.7% increase in VDR concentration (*p* = 0.025) compared to changes of −7.4% and 7.8%, respectively, in the placebo group.
Lagari et al., 2013, USA [117]	Older adults aged 65–95 years who were ambulatory, and community dwelling, mean age: 73.4 ± 6.4 years. *n* = 105 randomized, *n* = 86 participated in sub-study	400 IU or 2000 IU vitamin D_3_ daily	6 months	Physical performance (gait speed, timed sit-to-stand, single leg balance, gallon jug test, handgrip), body composition, levels of serum 25(OH)D, calcium, creatinine, and spot urine calcium	There was no improvement in physical performance for either group; however, supplementation was more effective in those with low baseline 25(OH)D levels (<30 ng·dL^−1^. The relative change in serum 25(OH)D (%) was positively associated to change in chair stand test score (5.1%, *p =* 0.033) and inversely associated with an increase in fat mass (*p =* 0.027).
Bauer et al., 2015, The Netherlands [118]	Older adults aged 65 years or older with mild to moderate physical limitations and low skeletal muscle index, mean age: 77.3 ± 6.7 and 78.1 ± 7.0 years for the experimental and placebo group, respectively. *n* = 380 were randomized, 302 completed all three study visits	Active control product (20 g whey protein, 3 g leucine, 9 g carbohydrate, 3 g fat, 800 IU vitamin D) *n* = 184, or isocaloric control, *n* = 196 consumed twice daily	13 weeks	Handgrip strength, SPPB, appendicular muscle mass, serum 25(OH)D concentrations	Handgrip strength increased by 0.79 kg in 13 weeks in the active group (*p* = 0.005), with no significant increase in the control. The SPPB scores increased in both groups (*p <* 0.001), with chair stand time improving more in the active group (*p* = 0.018). An estimated mean difference of 0.17 kg of appendicular muscle mass showed a significant increase over the placebo (*p <* 0.001).
Cangussu et al., 2015, Brazil [119]	Post-menopausal females aged 50–65 years, mean age: 55.6 ± 6.6 years. *n* = 160 randomized and included as intention to treat, *n* = 140 analyzed per protocol	1000 IU Vitamin D_3_ (*n* = 80) or identical placebo (*n* = 80)	9 months	Handgrip strength, Chair stand, lean body mass and fat mass assessed by DXA, serum 25(OH)D concentrations, creatinine, calcium, and parathyroid hormone levels	The supplementation group had a 45.4% increase in serum 25(OH)D levels at 9 months compared to the 18.5% decrease seen in the placebo group. Lean mass decreased 6.8% over 9 months only in the placebo group (*p* = 0.030). The supplementation group had a 25.3% increase in chair stand performance, whereas there were no changes in the placebo (*p* < 0.0001). Neither group had a change in handgrip strength.
* Apaydin et al., 2018, Turkey [120]	Postmenopausal females ages 50–68 years with vitamin D levels <20 ng·mL^−1^., mean age: 51.6 ± 5.8 and 51.58 ± 5.54 years in the daily and single dose groups, respectively. *n* = 60	800 IU daily (*n* = 32) or a single bolus of 300,000 IU (*n* = 28) of vitamin D_3_	3 months	Serum 25(OH)D concentrations, muscle strength of the quadriceps and hamstrings	There were no differences in muscle strength between groups; however, the daily dose group showed greater non-significant increases over time. Serum 25(OH)D levels increased in both groups but were higher in the single dose compared to the daily dose group.
** Vaes et al., 2018, The Netherlands [121]	Older adults aged 65 years or older with 25(OH)D levels between 20 and 50 nmol·L^−1^ and were considered frail or prefrail, mean age: 74.0 ± 6.0 years. *n* = 78	Daily supplements of 10 μg 25(OH)D_3_ (*n* = 26), 20 μg vitamin D_3_ (*n* = 24), or a placebo (*n* = 25)	6 months	Muscle strength (leg extension and flexion, handgrip strength), Physical performance (TUG, SPPB, postural sway), serum 25(OH)D concentrations, muscle fiber type from biopsies of the vastus lateralis, Body composition (appendicular lean mass, BMI)	The two supplementation groups had an increase in serum 25(OH)D levels over time, with the 10 μg 25(OH)D_3_ showing the greatest increase (*p* < 0.01). There were no differences between groups for muscle strength or performance
* Hajj et al., 2019, Lebanon [122]	Older adults who were pre-sarcopenic and vitamin D deficient, mean age: 73.3 ± 2.1 years. *n* = 128	vitamin D supplementation of 10,000 IU of vitamin D_3_ (*n* = 64) or a placebo (*n* = 64)	6 months	Handgrip strength, appendicular skeletal muscle mass, serum 25(OH)D concentrations	The supplement group had a greater change in serum 25(OH)D levels (10.13 to 27.98 ng·mL^−1^, *p* < 0.001) compared to the placebo (10.56 to 15.71 ng·mL^−1^, *p* < 0.001). Handgrip strength increased approximately 0.85 kg (*p* = 0.007), and appendicular skeletal muscle mass increased 0.65 kg (*p* = 0.001) at 6 months, whereas the placebo group had no differences over time.
Molmen et al., 2021, Norway [123]	Older adults with healthy lung function or diagnosed with COPD, ages 65–77, mean age: 68.0 ± 5.0 years. *n* = 95 enrolled, 78 completed	Vitamin D_3_ supplementation of 10,000 IU per day for two weeks and 2000 IU per day for the remainder (*n* = 34) or the study or a placebo (*n* = 44)	12 weeks of vitamin D supplementation only, followed by 13 weeks of supplementation + resistance training	One repetition maximum (1 RM) of leg extension and leg press, number of repetitions at 50% of 1 RM, isokinetic peak torque, VO_2_max, Wmax, sit-to-stand, 6 min step test, muscle thickness, leg lean mass, blood analysis for total testosterone, cortisol, growth hormone, IGF-1, SHBG, androstenedione, serum 25(OH)D concentrations, PTH, calcium, albumin, creatinine, creatine kinase AST, CRP, triglycerides, LDL, HDL, thyroid hormones, and iron status variables, and muscle fiber cross-sectional area and nuclei number from biopsies.	Overall, supplementation resulted in a 42 nmol·L^−1^ increase in serum 25(OH)D concentrations (*p* < 0.001) but did not lead to any additional improvement in training-related effects.
Youth					
** Ward et al., 2010, UK [124]	Post-menarchal females ages 12–14 years, mean age: 13.8 ± 0.6 years. *n* = 73, *n* = 72 completed intervention	4 doses per year of 150,000 IU vitamin D_2_ (*n* = 36) or placebo (*n* = 36)	12 months	Muscle force, power velocity, and jumping height during countermovement vertical jumps, grip strength, serum 25(OH)D concentrations, PTH, bone mineral density	Serum 25(OH)D concentrations increased by 12.3 nmol·L^−1^ (*p* < 0.001) in the intervention group while decreasing by −0.8 nmol·L^−1^ in the placebo group. Mean PTH decreased in both groups. There were no significant changes between groups for muscle parameters or bone mineral density.
Wright et al., 2018, USA [125]	Children age 9–13 years in the United States, mean age: 11.3 ± 1.2 years. *n* = 324	0, 400, 1000, 2000, or 4000 IU vitamin D_3_ per day	12 weeks	Serum 25(OH)D and 1,25(OH)2D concentrations, body composition (fat-free mass, fat mass, body fat percent, forearm and calf muscle cross-sectional area, muscle density, intramuscular adipose tissue, and handgrip strength.	Changes in muscle mass and strength over the 12 weeks were not related to changes in 25(OH)D, even with a 34.9% (*p <* 0.001) increase in serum 25(OH)D levels. However, increases in 25(OH)D were inversely associated with forearm intramuscular adipose tissue (r = −0.17, *p =* 0.029).
Mortensen et al., 2019, Denmark [126]	Children aged 4–8 years in Copenhagen, mean age: 6.6 ± 1.5 years. *n* = 117	10 μg·day^−1^ (*n* = 38), 20 μg·day^−1^ (*n* = 39), or a placebo (*n* = 40)	20 weeks	Handgrip strength. BMI, fat mass index, fat-free mass index, and serum 25(OH)D, IGF, and IGF-binding protein concentrations	At baseline, serum 25(OH)D concentrations were positively associated with handgrip strength (non-adjusted: β = 0.73–0.74, *p* = 0.005–0.006, adjusted: β = 0.38–0.39, *p* = 0.022–0.025), fat-free mass index (non-adjusted: β = (0.26, *p* = 0.001, adjusted: β = 0.19, *p =* 0.006), and IGF binding protein (β = 0.19, *p* = 0.010) in females. Levels of serum 25(OH)D decreased by 24.2 μg·day^−1^ in the placebo group and increased by 4.9–17.4 μg·day^−1^ (*p* < 0.001) in both intervention groups. There were no differences between groups after the intervention for handgrip strength or fat-free mass; however, IGF-1 was higher in the 20 μg·day^−1^ group compared to the placebo

* Denotes studies that specifically examined vitamin D deficient individuals. ** Denotes studies that specifically examined vitamin D insufficient individuals.

**Table 3:** Cross-sectional studies examining associations between anemia, iron status and muscle health.

Author, Year, Country	Study Participants	Measurements	Conclusions
Athletes			
DellaValle & Hass, 2012, USA [46]	Female rower athletes *n* = 165, *n* = 44 of rowers were identified as iron depleted without anemia (IDNA) [serum ferritin < 20.0 μg·L^−1^]; *n* = 16, as anemic (hemoglobin < 12.0 g·dL^−1^).	Physical performance of 48 nonanemic rowers (*n* = 24 normal, *n* = 24 depleted) was assessed during a 4 km rowing event (VO_2_max, time and energetic efficiency), ferritin, sTfR, and Hb levels	Rowers with IDNA had 0.3 L·min^−1^ lower VO_2_max and VO_2_peak (*p* = 0.02–0.03) and higher lactate concentration during a 4 km row assessment (*p* = 0.02). Relationships between iron status and endurance performance (VO_2_max, VO_2_peak, time, and energetic efficiency), between groups was dependent on the rowers’ training load. A positive correlation was present between VO_2_peak and serum ferritin (r = 0.29, *p =* 0.05), but no other measures of iron status with performance. IDNA may influence the training load of athletes affecting performance.
Tsai et al., 2019, Taiwan [170]	Males Taiwanese Military *n* = 3666	3000 m run test, 2 min sit-ups and 2 min push-up test, levels of Hb and hematocrit	After adjusting for age, service occupation, BMI, waist size, and blood pressure, mild anemic males were more likely to be the worst 10% performers in the 3000 m run test (Odds Ratios 1.47, *p* = 0.043). However, mild anemic males had a higher possibility to be the best 10% performers in the 2 min push-ups test (Odds Ratio 1.68, *p* = 0.001). There was no associations between anemic status and 2 min sit-up test.
Shoemaker et al., 2019, USA [171]	Male (*n* = 179; mean age 12.0 ± 2.1 years) and female (*n* = 70; mean age 12.0 ± 2.2 years) youth athletes	Athletic performance (vertical jump, broad jump, agility drill times, 20-yard dash time, power push up force), dietary intakes, levels of ferritin, sTfR, and Hb	Athletic performance was consistently related to Hb in males (r = 0.237–0.375, *p* < 0.001–0.05) and with sTfR (r = 0.521–0.649, *p* < 0.001–0.004) and iron intake (r = 0.397–0.568, *p =* 0.001–0.027) in females.
Older Adults			
Juárez-Cedillo et al., 2014, Mexico [172]	Older adults from the Study on Aging and Dementia, mean age by Hb quintiles: 71.7 ± 7.6, 70.8 ± 7.0, 71.6 ± 7.7, 69.7 ± 7.3, and 70.8 ± 7.4 years from lowest to highest Hb quintile, respectively. *n* = 1933	Levels of Hb, frailty status (weight loss, exhaustion, grip strength, and walking speed)	Greater risk of frailty was present in those with lower Hb concentrations, with concentrations of Hb of 10.5 and 11.5 g·dL^−1^ having greater likelihood of frailty (Odds Ratio 6.3 and 2.3) compared to concentrations of 15.0 g·dL^−1^ (Odds Ratio 0.81).
Kim et al., 2014, Korea [173]	Older adults in South Korea from the KNHANES IV study that were 60 years or older, mean age: males, 69.0 ± 6.3; females, 69.3 ± 6.4 years. *n* = 2332	Ferritin concentrations, HOMA-IR, sarcopenic status based on appendicular skeletal muscle mass	Ferritin concentrations were higher in the sarcopenic females compared to the non-sarcopenic females (70.7 vs. 85.4 ng·mL^−1^, *p* = 0.001). Appendicular skeletal muscle was negatively associated with ferritin concentrations (males; r = −0.111, *p =* 0.001, females; r = −0.104, *p* < 0.001).
Pires Corona et al., 2015, Brazil [174]	Older adults in Sao Paulo, Brazil, mean age 70.0 years. *n* = 1256	Levels of Hb, frailty status (weight loss, exhaustion, grip strength, and walking speed)	Mean Hb concentrations were lower in frail older adults compared to non-frail older adults (13.3 g·dL^−1^ vs. 14.3 g·dL^−1^, *p* < 0.001). Frail individuals were more likely to have lower Hb than non-frail individuals, independent of confounding health conditions (Odds Ratio 3.27, *p* < 0.001).
Moon et al., 2015, Korea [175]	Korean males 65 years or older from the KNHANES and young males not meeting study criteria included as a reference group, mean ages: 71.6 ± 5.0 and 30.7 ± 5.9 years, respectively. *n* = 1464	Levels of Hb, Skeletal Muscle Index	Low muscle mass was related to presence of anemia, independent of potential confounding factors (Odds Ratio 2.83).
Ruan et al., 2019, China, Ghana, India, Mexico, Russia, South Africa [176]	Older adults in China age 50 years or older that were part of the World Health Organization Study on Global Ageing and Adult Health, mean age 62.6 ± 0.2 ^#^ years. *n* = 13,175	Levels of Hb, frailty status	Presence of anemia was associated with frailty, in which for each 1 g·dL^−1^ increase in Hb concentration, there was a 4% decrease in the odds of frailty (Odds Ratio 0.96, *p* < 0.001).
Neidlein et al., 2021, Germany [177]	Hospitalized older adults aged 65 years or older, mean age: 81.4 ± 6.2 years. *n* = 224	Levels of ferritin, transferrin, iron, and Hb, CRP, handgrip strength, SPPB score, isometric leg extension strength. Iron supplementation protocol was recorded if provided during hospital stay	In those with iron deficiency, frailty scores were higher (4 vs. 3, *p* < 0.05), Hb was lower (males; 11.1 g·dL^−1^ vs. 12.4 g·dL^−1^, *p <* 0.01, females; 10.7 g·dL^−1^ vs. 11.7 g·dL^−1^, *p* < 0.001), and CRP was higher (3.2 mg·dL^−1^ vs. 1.9 g·dL^−1^, *p* < 0.01). There were no differences in muscle strength and function parameters between those with were iron deficient or non-iron deficient. However, a positive association was observed between Hb and handgrip strength at baseline and at hospital discharge in those with iron deficiency (β = 0.242–0.641, *p =* 0.020–0.039).
Youth			
Arsenault et al., 2011, Colombia [169]	Youth aged 5–12 years in Colombia. *n* = 1945	Standing long jump, 36 m shuttle run, and levels of Hb, ferritin, vitamin B12, complete blood count, CRP, erythrocyte folate	There were no differences in performance measurements between anemic and non-anemic youth. Females with low ferritin had 0.6 s slower performance on the shuttle run than females with normal ferritin levels *(p* = 0.020). Males with low ferritin had 7 cm lower jump scores than those with normal ferritin (*p* = 0.030).
Gracia-Marco et al., 2012, Sweden, Greece, Italy, Spain, Hungary, Belgium, France, Germany, Austria [99]	Adolescents aged 12.5–17.5 years old across Europe that completed the blood sample analysis as part of the HELENA-CSS study. *n* = 1089; males, *n* = 509, females, *n* = 580	Standing long jump, 20 m shuttle run to estimate VO_2_max, red blood cell parameters, biomarkers of iron status (sTfR and ferritin), other micronutrients (vitamins A, E, C, B6, and B12, folate, and serum 25(OH)D) concentrations	Concentrations of Hb was positively associated with estimated VO_2_max (from 20 m shuttle run) in male adolescents (β = 0.192, *p* = 0.002).

# Indicates Standard Error.

**Table 4:** Experimental studies examining the effects of iron supplementation on muscle health.

Author, Year, Country	Study Participants	Supplemental Iron Treatment	Duration	Measurements	Conclusions
Athletes					
DellaValle & Hass, 2014, USA [179]	31 Rowers	Treatment group: 100 mg·d^−1^ FeSO4 (*n* = 15) Placebo: (*n* = 16)	6-weeks	Iron status (Hb, iron ferritin, transferrin receptor), body composition, performance (4 km time trial, VO_2_max, energetic efficiency, and blood lactate)	Treatment group showed a 0.3 g·dL^−1^ improvement in Hb (*p* = 0.04), slower lactate response during the 4 km time trial, and (12.2 nmol·L^−1^ vs. 11.4 nmol·L^−1^ at post-treatment compared to placebo, *p* < 0.001), and a 4.3 kcal higher energy expenditure (*p* = 0.03) post-treatment.
Garvican et al., 2014, Australia [180]	27 distance runners with low or suboptimal iron status	Intravenous (IV) iron (550 ± 171 mg for low iron status, 375 ± 39 mg for suboptimal iron status) or oral supplementation of one (low) or two (suboptimal) tablets of 305 mg ferrous sulfate and 105 mg elemental iron) daily	6-weeks	Iron status (Hb, sTfR, ferritin, erythropoietin, transferrin, Hb mass) and performance (VO_2_max, lactate threshold, running economy)	Both IV and oral supplementation showed a 83.7–417.5% increase ferritin at 6 and 8 weeks and a −5.6–−9.9% decrease in sTfR concentrations. VO_2_max increased, with a more profound increase in runners with low iron status (1.2–3.3 mL·kg^−1^·min^−1^).
Mielgo-Ayuso et al., 2015, Spain [181]	22 Elite female volleyball players (27.0 ± 5.6 years)	Treatment group: 325 mg·d^−1^ ferrous sulphate daily (*n* = 11) Placebo: (*n* = 11)	11-weeks	Iron status (serum iron, ferritin, transferrin saturation index, Hb), and strength (bench press, military press, half-squat, power clean, clean and jerk, and pull-over)	Treatment group showed significantly greater levels of serum iron, ferritin, transferrin saturation index, Hb compared to placebo group. Improvements in bench press (38.2 to 43.1 kg) clean and jerk (27.7 to 35.1 kg), power clean (33.3 to 35.1 kg), and total mean strength (35.2 to 41.9 kg occurred after the 11 weeks in the treatment group compared to placebo group (*p* < 0.05).

## Data Availability

Not applicable.
